# Verifying the fully “Laplacianised” posterior Naïve Bayesian approach and more

**DOI:** 10.1186/s13321-015-0075-5

**Published:** 2015-06-12

**Authors:** Hamse Y Mussa, David Marcus, John B O Mitchell, Robert C Glen

**Affiliations:** Department of Chemistry, Centre for Molecular Informatics, Lensfield Road, Cambridge, England CB2 1EW UK; European Bioinformatics Institute (EMBL-EBI), European Molecular Biology Laboratory, Wellcome Trust Genome Campus, Hinxton, Cambridge, England CB10 1SD UK; EaStCHEM School of Chemistry and Biomedical Sciences Research Complex, University of St Andrews, North Haugh, St Andrews, Scotland KY16 9ST UK

**Keywords:** Classification, Naïve Bayes, Tapering, Features

## Abstract

**Background:**

In a recent paper, Mussa, Mitchell and Glen (MMG) have mathematically demonstrated that the “Laplacian Corrected Modified Naïve Bayes” (LCMNB) algorithm can be viewed as a variant of the so-called Standard Naïve Bayes (SNB) scheme, whereby the role played by absence of compound features in classifying/assigning the compound to its appropriate class is ignored. MMG have also proffered guidelines regarding the conditions under which this omission may hold. Utilising three data sets, the present paper examines the validity of these guidelines in practice. The paper also extends MMG’s work and introduces a new version of the SNB classifier: “Tapered Naïve Bayes” (TNB). TNB does not discard the role of absence of a feature out of hand, nor does it fully consider its role. Hence, TNB encapsulates both SNB and LCMNB.

**Results:**

LCMNB, SNB and TNB performed differently on classifying 4,658, 5,031 and 1,149 ligands (all chosen from the ChEMBL Database) distributed over 31 enzymes, 23 membrane receptors, and one ion-channel, four transporters and one transcription factor as their target proteins. When the number of features utilised was equal to or smaller than the “optimal” number of features for a given data set, SNB classifiers systematically gave better classification results than those yielded by LCMNB classifiers. The opposite was true when the number of features employed was markedly larger than the “optimal” number of features for this data set. Nonetheless, these LCMNB performances were worse than the classification performance achieved by SNB when the “optimal” number of features for the data set was utilised. TNB classifiers systematically outperformed both SNB and LCMNB classifiers.

**Conclusions:**

The classification results obtained in this study concur with the mathematical based guidelines given in MMG’s paper—that is, ignoring the role of absence of a feature out of hand does not necessarily improve classification performance of the SNB approach; if anything, it could make the performance of the SNB method worse. The results obtained also lend support to the rationale, on which the TNB algorithm rests: handled judiciously, taking into account absence of features can enhance (not impair) the discriminatory classification power of the SNB approach.

## Background

Pattern classification techniques are indispensable in cheminformatics. For example, a cheminformatian might be interested in knowing whether: a particular small compound (ligand) is capable of inducing a desirable biological effect on a specific target protein [1, 2]; an enzyme catalyses a certain chemical reaction, or a catalytic mechanism of an enzyme is appropriate for a chemical reaction [3]; a substructure of a substrate is a site of metabolism [4]; a ligand is structurally similar to a reference set of ligands known to possess desirable physical, chemical and biological properties [2, 5, 6]; a protein is a potential target for a given ligand [2, 7]; etc.

In all these examples, the underlying task can be viewed as a classification problem hinging on the assumption that there are inherently underlying characteristic patterns in the proteins, ligands, substrates, etc. It should, therefore, come as no surprise that a considerable body of literature exists highlighting and expounding on the important role pattern classification methods have in cheminformatics—summarised in many articles, such as these recent Refs. [8–10].

Formally, a pattern classification problem deals with the optimal assignment of an object to one of $J$ predefined classes/categories, $(\omega _1,\omega _2,\ldots,\omega _J)$, whereby it is assumed that the object is adequately (or even better, uniquely) characterized by *L* features x$_{l}$, with $l=1,2,\ldots,L$. Typically, the object (or simply the pattern) is represented by an *L*-dimensional vector **x**, whose elements x$_{l}$ are discriminating features that ideally codify the pattern. Thus, mathematically, the classification problem may be cast as establishing a mapping from pattern feature space $\mathcal {X}$, in which pattern vectors **x** reside, into class space $\Omega$ comprising our predefined set of classes:

1 $$\begin{aligned} f:\mathcal{{X}}\rightarrow \Omega \end{aligned}$$

such that any pattern **x**$\in \mathcal {X}$ can be assigned to its appropriate class/label $\omega _j$, where $\omega _j\in \Omega = (\omega _1,\omega _2,\ldots,\omega _J)$.

To this end, it is desirable to identify *L* underlying characteristics of the pattern to render the mapping in Eq. 1 a simple look-up table. In practice, however, identifying *L* relevant features to classify new patterns without classification errors is generally impossible. In this scenario, the classification problem becomes finding a mapping between $\mathcal {X}$ and $\Omega$ that minimises the misclassification rate [11–15]. One way of achieving this objective is to treat both the pattern vector **x** and $\omega _{j}$ as random variables; compute the class $\omega _{j}$ probabilities for a given pattern **x**, $p(\omega _{j}|\mathbf{x})$; and then assign **x**$\in \mathcal {X}$ to the class $\omega _{j}\in \Omega$ for which the $p(\omega _{j}|\mathbf{x})$ value is maximum [11–14]. (In the last step it is being assumed that all misclassification errors are equally bad [11, 13, 14].)

More often than not, $p(\omega _{j}|\mathbf{x})$ is unknown. Instead, one has access to a representative data set comprising $N$ prototype pairs, $\mathcal{D}=\{(\mathbf{x}_{i},y_{i})\}^{N}_{i=1}$, drawn from the joint probability density function $p(\omega _{j},\mathbf{x})$ over $\omega _{j}$ and **x**, where $y_{i}\in \{\omega _{j}\}^{J}_{j=1}$ and **x**$_{i}$ denotes pattern *i* whose class label is y$_{i}$. This means Eq. 1 can amount to approximating $p(\omega _{j}|\mathbf{x})$ from $\mathcal{D}$. In other words, the pattern classification problem now becomes a statistical problem. In any event, in practice, it is not always an easy task to estimate $p(\omega _{j}|\mathbf{x})$ nor $p(\omega _{j},\mathbf{x})$ from $\mathcal{D}$ (the so-called training set) [5, 11, 13–15]. However, probability rules and Bayes’ theorem allow one to modularize the problem and estimate $p(\omega _{j}|\mathbf{x})$ in terms of probability density functions that we may have a better chance of being able to estimate utilising $\mathcal{D}$. To this end, $p(\omega _{j}|\mathbf{x})$ is defined as [5, 11, 13],

2 $$\begin{aligned} p(\omega _{j}|\mathbf{x}) = \frac{p(\omega _{j},\mathbf{x})}{p(\mathbf{x})} = \frac{p(\mathbf{x}|\omega _{j})p(\omega _{j})}{\sum ^{M}_{j=1}p(\mathbf{x}|\omega _{j})p(\omega _{j})} \end{aligned}$$

where estimating $p(\mathbf{x}|\omega _{j})$ and $p(\omega _{j})$ from the available training set can be easier than estimating $p(\omega _{j}|\mathbf{x})$—or for that matter, $p(\omega _{j},\mathbf{x})$ and $p(\mathbf{x})$—directly from $\mathcal{D}$. In Bayesian statistics settings, $p(\omega _{j})$ is referred to as the class prior probability function, which is the probability that a member of class $\omega _{j}$ will occur. The function $p(\mathbf{x}|\omega _{j})$ is called the class-conditional probability density function, i.e., the probability density of observing pattern **x** given that **x** is a member of class $\omega _{j}$. The denominator term in Eq. 2 is often called the “evidence”, “prior predictive”, “marginal likelihood”, and others. As far as this paper is concerned, suffice it to say this term can be viewed as a normalisation factor.

Typically, $p(\omega _{j})$ is assumed to be uniform, i.e., $p(\omega _{j})=\frac{1}{J}$, where *J* is as defined before. If, however, there is convincing evidence that the number of pairs $(\mathbf{x}_{i},\omega _{j})$ per class in the training data set is an indication of the importance of that class, then a sensible approximation of $p(\omega _{j})$ can be

3 $$\begin{aligned} p(\omega _{j}) = \frac{N_{\omega _{j}}}{N} \end{aligned}$$

where $N_{\omega _{j}}$ denotes the number of patterns in $\mathcal{D}$ that belong to class $\omega _{j}$, and *N* is as described before.

Direct computation of $p(\mathbf{x}|\omega _{j})$ from the training data set is not so straightforward as estimating $p(\omega _{j})$ [5, 11–15], especially for *L* ≥ 100, as modeling a joint probability distribution that captures the relationship among the *L* features $x_{l}$ given $\omega _{j}$ can become quite involved. The main difficulty is that the required size of $\mathcal{D}$ increases exponentially with *L* [11, 16].

Over the past six decades, a plethora of methods have been proposed to estimate $p(\mathbf{x}|\omega _{j})$ from a given training data set [13, 17, 18]. In this paper we are concerned with one particular method that is widely utilised in cheminformatics (and elsewhere): the so-called Naïve Bayes approach [5, 11, 13, 15]. It is based on the simplistic assumption that the *L* features $x_{l}$ are statistically independent given $\omega _{j}$. This Naïve assumption (hence: the name “Naïve Bayes”) significantly mitigates the difficulty of estimating the fully joint class-conditional probability density function $p(\mathbf{x}|\omega _{j})$ to that of estimating $L$ statistically independent class-conditional univariate probability density functions $p(x_{l}|\omega _{j})$. In other words, this simplistic scheme ignores possible dependencies, i.e., correlations, among the *L* pattern features $x_{l}$ given $\omega _{j}$, and approximates $p(\mathbf{x}|\omega _{j})$ as a product of *L* class-conditional univariate density functions $p(x_{l}|\omega _{j})$:

4 $$\begin{aligned} p(\mathbf{x}|\omega _{j}) = \Pi ^{L}_{l=1}p(x_{l}|\omega _{j}) \end{aligned}$$

A decade ago, Xia et al. [19] further “simplified” this Naïve scheme itself, in particular when the features are binary, i.e., $x_{l}$ = 0 or 1 denoting an absence or presence of feature $x_{l}$ in the pattern vector **x**, respectively. Instead of the conventional $p(x_{l}|\omega _{j})$, the authors estimated $p(\omega _{j}|x_{l})$, though—as we will see shortly, the two functions are related.

Using clever heuristic arguments, these authors deemed absence of features, i.e., $x_{l}=0$, unimportant or even problematic for estimating $p(\omega _{j}|\mathbf{x})$, the probability density function that we are ultimately concerned with. That is to say, in their approach, which they termed the “Laplacian Corrected Modified Naïve Bayes” (LCMNB), only $p(\omega _{j}|x_{l})$ for $x_{l}=1$ were judged pertinent and relevant for estimating $p(\omega _{j}|\mathbf{x})$. In passing, there are many interesting and useful aspects of LCMNB [5–7, 19], with which we are not concerned in this paper. Since its introduction, the LCMNB approach has been employed in cheminformatics for both in silico ligand-based virtual screening and target protein prediction approaches [6, 7, 20–22].

Before proceeding further, it is worth noting that LCMNB should not be confused with the so-called Multinomial Naïve Bayes, another variant of the Naïve Bayes algorithm [23]. LCMNB is closely related to the Naïve Bayes variant called the Bernoulli Naïve Bayes [23], henceforth referred to as the Standard Naïve Bayes (SNB). In a recent paper, henceforth referred to as MMG, Mussa et al. [5] have demonstrated the relationship between the LCMNB and SNB algorithms. In MMG it has been explicated that LCMNB can be viewed as an instance of SNB, under certain conditions. In broader terms, MMG questioned, albeit tacitly, whether it would be justifiable to discard absence of a feature out of hand.

Using three data sets, the present paper examines whether these theory based conditions and questions—regarding the application of LCMNB based classifiers—have any practical use or are just merely a mathematical exercise and curiosity.

In this study, we also extend the work presented in MMG to introduce a novel classification scheme termed “Tapered Naïve Bayes” (TNB). In TNB, unlike LCMNB and SNB, the absence of a feature is neither completely discarded nor is it fully considered. In other words, TNB subsumes both SNB and LCMNB as illustrated in the following section. In that section, we set the scene and briefly describe the SNB, LCMNB and TNB algorithms. The section also presents a description of the three data sets employed to construct, test and compare classifiers based on SNB, LCMNB and TNB. Our findings and analyses, and concluding remarks are given in "Results and conclusion", respectively.

In the following discussions, **x**, ${\omega }_{j}$, and $\mathbf{x}_{l}$ denote both random variables and their instantiations. Again to keep notations less cluttered, the estimated density functions and their corresponding true density functions are not distinguished. We follow—in line with the current trend in machine learning and statistics—the convenient, although strictly not accurate, practice of using the term “density” for both a discrete random variable’s probability function and for the probability function of a continuous random variable [24]. The terms “category”, “class”, “label”, and “class label” are used interchangeably.

Finally, in this work, we are only concerned with patterns represented by binary feature vectors **x** residing in binary feature space: **x** $\in \mathcal{{X}} =\{0,1\}^{L}$, i.e., $x_{l}\in ~\{0,1\}$.

## Methods

For completeness, the mathematical relationship between the SNB and LCMNB approaches is described first and then the conditions, under which LCMNB can be considered as a variant of SNB, are stated. This material has been covered in great detail in MMG. Finally we give the formulae that describe the TNB algorithm.

In "Background", it has been noted that $p(\omega _{j}|\mathbf{x})$ can be expressed in terms of $p(\mathbf{x}|\omega _{j})$ and $p(\omega _{j})$ that can be estimated from a given training data set. One way of mitigating the difficulty of computing $p(\mathbf{x}|\omega _{j})$ has also been discussed: introducing the so-called Naïve Bayes assumption, which allows one to approximate $p(\mathbf{x}|\omega _{j})$ as a product of *L* class-conditional univariate density functions, see Eq. 4.

### Bernoulli Naïve Bayes: standard Naïve Bayes

Since we are concerned with patterns whose features are binary, i.e., $x_{l}$ = 0 or 1, Eq. 4 can be expressed in a more compact form:

5 $$\begin{aligned} p(\mathbf{x}|\omega _{j})&= \Pi ^{L}_{l=1}\big [p(x_{l}=1|\omega _{j})\big ]^{x_{l}}\big [p(x_{l}=0|\omega _{j})\big ]^{1-x_{l}}\nonumber \\&= \Pi ^{L}_{l=1}\left [\frac{p(x_{l}=1)p(\omega _{j}|x_{l}=1)}{p(\omega _{j})}\right ]^{x_{l}}\left [\frac{p(x_{l}=0)p(\omega _{j}|x_{l}=0)}{p(\omega _{j})}\right ]^{1-x_{l}}, \end{aligned}$$

whereby, in the second line on the right hand side of Eq. 5, we made use of Bayes’ theorem: $p(x_{l}|\omega _{j}) = \frac{p(\omega _{l}|x_{l})p(x_{l})}{p(\omega _{j})}$, with $p(\omega _{j})$ and $p(x_{l}|\omega _{j})$ being as defined before, whereas $p(x_{l}) = \sum ^{J}_{j=1}p(x_{l}|\omega _{j})p(\omega _{j})$ with *J* denoting the total number of classes. Inserting the second line of Eq. 5 into Eq. 2 and then taking logarithm of the resultant equation, we obtain

6 $$\begin{aligned} \log p(\omega _j|\mathbf{x})_{SNB}&= {} \sum ^{L}_{l=1}x_{l}\log p(\omega _j|x_{l}=1) + \sum ^{L}_{l=1}(1-x_{l})\log p(\omega _j|x_{l}=0) - (L-1)\log p(\omega _j) \nonumber \\&\quad + {} \sum ^{L}_{l=1}x_{l}\log p(x_{l}=1) + \sum ^{L}_{l=1}(1-x_{l}) \log p(x_{l}=0) - \log p(\mathbf{x}) \end{aligned}$$

where all the terms and variables are as defined before, and the subscript “SNB” in $\log p(\omega _j|\mathbf{x})_{SNB}$ indicates that the equation represents the SNB algorithm. The third line of Eq. 6 can be ignored as the terms in this line are class independent and play no role in classifying the pattern **x**; for more technical details, the reader is referred to ref [5]. Thus, in practice, Eq. 6 reduces to

7 $$\begin{aligned} \log p(\omega _i|\mathbf{x})_{SNB}=\sum ^{L}_{l=1}x_{l}\log p(\omega _j|x_l=1) + \sum ^{L}_{l=1}(1-x_{l})\log p(\omega _j|x_l=0) - (L-1)\log p(\omega _j) \end{aligned}$$

(Note that it is better to perform the computation by adding logarithms of probabilities because multiplying many conditional probabilities can lead to a floating point underflow.)

As a side, but important, note: recall that, when one utilises a 0/1 function (i.e., all misclassification errors are assumed to be equally bad), Bayes’ decision rule assigns $\mathbf{x}$ to class $\omega _{j}$ if [11, 15]

8 $$\begin{aligned} \log p(\omega _{j}|\mathbf{x})_{SNB} = \arg \!\max _{k}\log p(\omega _{k}|\mathbf{x})_{SNB} \end{aligned}$$

In this scenario, Eq. 7 combined with Eq. 8 constitutes the SNB classifier [5].

### Laplacian corrected modified Naïve Bayes

Using heuristic arguments, Xia et al. [19] estimated $p(\omega _{j}|\mathbf{x})$ in terms of only $p(\omega _{j}|x_{l}=1)$ and $p(\omega _{j})$, and then took the logarithm of the estimated $p(\omega _{j}|\mathbf{x})$ obtaining

9 $$\begin{aligned} \log p(\omega _j|\mathbf{x})_{LCMNB}=\sum ^{L}_{l=1}x_{l}\log p(\omega _j|x=1) - (L-1)\log p(\omega _j) \end{aligned}$$

where the terms and variables are as defined before, and and the subscript “LCMNB” in $\log p(\omega _j|\mathbf{x})_{LCMNB}$ indicates that the equation is for the LCMNB algorithm. Once again, Eq. 9 and the decision rule that assigns $\mathbf{x}$ to class $\omega _{j}$ if

10 $$\begin{aligned} \log p(\omega _{j}|\mathbf{x})_{LCMNB} = \arg \!\max _{k}\log p(\omega _{k}|\mathbf{x})_{LCMNB} \end{aligned}$$

define the LCMNB classifier [19].

Clearly Eqs. 7 and 9 are the same with one notable difference: the term $\sum ^{L}_{l=1}(1-x_{l})\log p(\omega _j|x_l=0)$ is missing in Eq. 9, which is clearly the term concerned with absence of features $x_{l}$ in Eq. 7. The omission of this term from Eq. 9 embodies the central tenet of the SNB simplification proposed by Xia *et al.*

### The conditions

Now we come to the nub of this paper: testing the conditions under which LCMNB can be considered as a simpler version of SNB [5] in principle, or in practice (or both).

It is clear from Eqs. 7 and 9 that ignoring

$$\begin{aligned} \sum ^{L}_{l=1}(1-x_{l})\log p(\omega _j|x_l=0) \end{aligned}$$

may not matter so long as $\log p(\omega _j|\mathbf{x})_{LCMNB}> \log p(\omega _k|\mathbf{x})_{LCMNB}$ whenever $\log p(\omega _{j}|\mathbf{x})_{SNB}> \log p(\omega _{k}|\mathbf{x})_{SNB}$ for any given pattern **x**. For all practical purposes, this requirement may amount to meeting the following two conditions:;

1. For any pattern vector **x**, the value of $\sum ^{L}_{l=1}(1-x_{l})\log p(\omega _j|x_l=0)$ is the same (or can be made so) in all classes;
2. For any pattern vector **x**
  $$\begin{aligned} \left |\sum ^{L}_{l=1}x_{l}\log p(\omega _j|x_l=1)\right |\gg \left |\sum ^{L}_{l=1}(1-x_{l})\log p(\omega _j|x_l=0)\right | \end{aligned}$$
  in all classes.

Naturally it raises the question of whether LCMNB can perform better than SNB even if Conditions 1 and 2 do not hold. In this paper, it is sought to answer this question as well.

### Tapered Naïve Bayes

Equations 7 and 9 are written in a suggestive manner, such that the two equations can be combined into a single equation:

11 $$\begin{aligned} \log p(\omega _j|\mathbf{x})_{TNB}=\sum ^{L}_{l=1}x_{l}\log p(\omega _j|x_l =1) + \lambda \sum ^{L}_{l=1}(1-x_{l})\log p(\omega _j|x_l=0) -(L-1)\log p(\omega _j) \end{aligned}$$

where $\lambda$ is a parameter. By setting $\lambda$ to 1 or 0, we recover SNB or LCMNB, respectively. This equation combined with the Bayes’ decision rule defined in Eq. 8—mutatis mutandis—constitutes the “Tapered Naïve Bayes” algorithm.

Clearly $\log p(\omega _j|\mathbf{x})_{TNB}$ are discriminant functions linear in the **x** [11, Chapter 2; 25]. In TNB, unlike SNB and LCMNB, these discriminant functions can be tuned to maximize the classification ability of the TNB based classifiers by tweaking the value of the $\lambda$ parameter in the interval [0,1]. Confining the value of $\lambda$ to the range $0\le \lambda \le 1$ means the parameter attenuates/tapers the contribution of the second term in Eq. 11 to estimating $\log p(\omega _j|\mathbf{x})$—hence, the acronym “Tapered Naïve Bayes”. The reason why the $\lambda$ value is being confined to the interval [0,1] is touched upon in the following paragraph. A full mathematical description of TNB will be given elsewhere.

One particular consequence of the simplistic assumption (SA) that makes the central tenet of the Naïve Bayes approach is that the SNB algorithm becomes intrinsically simple with high bias (but low variance) in its probability density estimates [26]. This bias increases with the value of *L* because the larger the value of *L* is the higher the chance of the *L* features becoming correlated. The fundamental reason for this increase of the chance of correlation among features is that in a high-dimensional feature vector there is the potential of many of its components being zero. That is to say, x$_{l}$ is more likely to assume 0 instead of 1 [19] which can lead to high correlation among features, which in turn can obviously render SA untenable—i.e., the Naive Bayes model severely misrepresents the data. This is the reason why, in our context, attenuation (not amplification) of the second term in Eq. 11 is required. This the main reason for limiting the allowed value of the $\lambda$ parameter to [0,1].

Of course, feature selection may help to address the bias problem—attributable to the contributions from absence of features—by removing the irrelevant and redundant features and in doing so reduce the size of the feature space, which in turn may decrease the chance of the features becoming correlated. However, this is a slightly different issue as LCMNB was arguably concerned with reducing the chance of correlations among features by simply discarding contributions from absence of features to the estimation of $p(\omega _{j}|\mathbf{x})$.

In any event, in the light of the preceding discussion, the LCMNB algorithm can clearly now be viewed as a severely penalised SNB algorithm.

Before we embark on testing the validity of Conditions (1) and (2), and also answer the question raised in "The conditions", we describe how to compute $p(\omega _{j}|x_{l}=0)$ and $p(\omega _{j}|x_{l}=1)$. We also outline the performance measures and statistics tests utilised to compare the three methods: TNB, SNB and LCMNB.

### Implementation and computation details

In this work the estimators of $p(\omega _j|x_l=1)$ and $p(\omega _j|x_l= 0)$ were computed using the following equations:

12 $$\begin{aligned} p(\omega _j|x_{l}=1) =\frac{p(x_{l}=1|\omega _{j})p(\omega _{j})}{p(x_{l}=1)}, \end{aligned}$$

where

13 $$\begin{aligned} p(x_{l}=1|\omega _{j}) =\frac{N_{lj} + \alpha _{j}}{N_{\omega _{j}}+\alpha _{j}+\beta _{j}}; p(\omega _{j})= \frac{N_{\omega _{j}}}{N} \end{aligned}$$

14 $$\begin{aligned} p(\omega _j|x_{l}=0) =\frac{p(x_{l}=0|\omega _{j})p(\omega _{j})}{p(x_{l}=0)}, \end{aligned}$$

where

15 $$\begin{aligned} p(x_{l}=0|\omega _{j}) = 1 - \frac{N_{lj} + \alpha _{j}}{N_{\omega _{j}}+\alpha _{j}+\beta _{j}}; p(\omega _{j})= \frac{N_{\omega _{j}}}{N} \end{aligned}$$

Both in Eqs. 12 and 14: $p(x_{l}) = \sum ^{J}_{j=1}p(x_{l}|\omega _j)p(\omega _{j})$. $N_{lj}$ denotes the number of times feature x$_{l}$ is present in class $\omega _{j}$, i.e., $x_l = 1$. The variables $\alpha _{j}$ and $\beta _{j}$ are *Beta* distribution parameters [5], both were set to 1; $N_{\omega _{j}}$, *N* and *J* are as described before.

### Data set

Bioactivity data were extracted from the ChEMBL17 database [27] for us to test: (a) the conditions under which SNB and LCMNB are equivalent, (b) whether LCMNB can yield better classification performance than SNB, and (c) the validity of the concept, on which the TNB algorithm, is based.

At the time accessed, the database comprised more than 1.3 million annotated compounds and more than 12 million bioactivity records covering 9,356 targets. To obtain the appropriate data points for our objective, we prioritised targets with the highest number of small ligands (≤1 kDa) annotated with *IC50* or *Ki* inhibitory binding values on single human proteins with high confidence scores of 9. Duplicates were removed by comparing the first level (non-stereochemistry) value of the *InChI* keys of each compound and then retaining the lowest annotated value in cases were more than one value was measured for the same compound. Since different targets have different activity value ranges, the minimal activity threshold to locate an active set was computed as the average of the negative logarithmic activity values (i.e., ${{-}{\log}} {Ki}$ or ${-}{\log} {IC50}$) plus one standard deviation above that activity value for each target. In cases where both *IC50* and *Ki* values were measured for the same target, we retained only the values sampled from the most abundant measured type in order to avoid mixing *IC50* and *Ki* values. The top 60 targets with the largest number of active ligands per target were then compiled and prepared for fingerprints calculations. ChemAxon’s Standardizer software [28] was utilised to remove fragments such as salts and ions; and explicit hydrogen atoms, and neutralising their structures.

This resulted in 10,838 small molecules (ligands) annotated over 60 target proteins. A closer look at this dataset revealed that it consisted of: 4,658 ligands annotated over 31 enzymes, Table 1; 5,031 ligands distributed over 23 membrane receptors, Table 2; and 1,149 ligands annotated over four transporters, one ion-channel and one transcription factor, Table 3. It was these three datasets that were utilised in this study.

**Table 1:** Enzyme data set: comprising 4,658 ligands to classify according to the enzyme they inhibit

Activity class	Target ID	No. of active compounds
Vascular endothelial growth factor 2	10980	268
Carbonic anhydrase II	15	262
11-beta-hydroxysteroid dehydrogenase 1	11489	233
Carbonic anhydrase I	10193	228
Beta-secretase 1	12252	212
Dipeptidyl peptidase IV	11140	208
Epidermal growth factor erbB1	9	202
MAP kinase p38 alpha	10188	197
Carbonic anhydrase IX	12952	184
Cyclooxygenase-2	194	180
Acetylcholinesterase	93	158
Coagulation factor X	126	156
Histone deacetylase 1	12697	156
Monoamine oxidase B	104	148
Thrombin	11	147
Renin	11225	139
Epoxide hydratase	11727	134
Matrix metalloproteinase 13	11024	125
Cathepsin K	10495	116
Cathepsin S	11534	112
Matrix metalloproteinase-2	13001	112
Protein-tyrosine phosphatase 1B	13061	110
Serine–threonine-protein kinase AKT	12666	109
Butyrylcholinesterase	10532	104
Cytochrome P450 19A1	65	102
Receptor protein-tyrosine kinase erbB2	188	99
Tyrosine-protein kinase SRC	10434	98
Hepatocyte growth factor receptor	11451	94
Matrix metalloproteinase-1	13000	91
Glycogen synthase kinase-3 beta	10197	89
Carbonic anhydrase XII	12209	85

Columns 1, 2 and 3 denote the protein, the protein identifier (ID) in our dataset and the number of ligands reported for each protein, respectively.

### Compound fingerprints

Extended Connectivity Fingerprints (ECFP) were calculated in RDKit using a Python based script to generate fixed-length ECFP4 binary fingerprints with a length of 1,024 bits—counting each bit once [29].

**Table 2:** Membrane-receptor data set: comprising 5,031 ligands to classify according to the biological activity they induce on these membrane receptors

Activity class	Target ID	No. of active compounds
Adenosine A2a receptor	252	424
Adenosine A3 receptor	280	356
Adenosine A1 receptor	114	322
Cannabinoid CB2 receptor	259	319
Histamine H3 receptor	10280	314
Cannabinoid CB1 receptor	87	304
Dopamine D2 receptor	72	281
Mu opioid receptor	129	269
Kappa opioid receptor	137	244
Delta opioid receptor	136	223
Melanocortin receptor 4	10142	220
Serotonin 1a (5-HT1a) receptor	51	215
Dopamine D3 receptor	130	213
Melanin-concentrating hormone receptor 1	19905	206
Serotonin 6 (5-HT6) receptor	10627	173
Serotonin 2a (5-HT2a) receptor	107	155
C-C chemokine receptor type 2	11575	150
Adenosine A2b receptor	278	136
G protein-coupled receptor 44	20174	117
Serotonin 2c (5-HT2c) receptor	108	114
Histamine H4 receptor	11290	96
C-C chemokine receptor type 5	10580	91
Nociceptin receptor	138	89

Columns 1, 2 and 3 denote the protein, the protein identifier (ID) in our dataset and the number of ligands reported for each protein, respectively.

The compounds were put together in one dataset and duplicated structures reported as being active against more than one target were removed in order to have unique active compounds on each target class. All the fingerprints were then read and binary values were set to the value 1 if the fingerprint was present in a compound and 0 if it was absent. The total number of fingerprints is set by the numbers of fingerprints in each dataset to avoid columns with 0 values for all compounds in the dataset. This gave a fingerprint of 1’s and 0’s, with an string length of 23,324, to represent each ligand in our dataset.

**Table 3:** Mixed class data set: comprising 1,149 ligands to classify according to the biological activity they induce on these transporters, transcription factor and ion-channel

Activity class	Target ID	No of active compounds
Serotonin transporter	121	222
Norepinephrine transporter	100	146
Dopamine transporter	155	136
Sodium/glucose cotransporter 2	20092	102
hERG	165	448
Peroxisome proliferator-activated receptor gamma	133	95

Columns 1, 2 and 3 denote the protein, the protein identifier (ID) in our dataset and the number of ligands reported for each protein, respectively.

In the context discussed in the "Background" and "Methods" sections: ligands in these datasets are the patterns **x**; the fingerprint denotes feature $x_{l}$, while *L* = 23,324; proteins denote the classes (class labels) $\omega _{j}$, i.e., *J* = 60; *N* is 4,658 (for the enzymes dataset), 5,031 (for the receptors dataset), and 1,149 (for transporters, ion-channel and transcription factor dataset, henceforth referred to as the mixed dataset).

Copies of the source code and the data sets utilised in this work can be obtained by sending a request to mussax021@gmail.com.

#### Model constructions and evaluation measures

A mutual information method (MIM) [30–32] was utilised to compute the pertinence of feature x$_{l}$ for pattern classification. MIM basically measures how much relevant information feature x$_{l}$ contributes to making the correct classification decision on a pattern belonging to class $\omega _j.$ Ideally the bigger this information (the so-called mutual information between feature x$_{l}$ and class $\omega _{j}$) the more useful the feature becomes for accurately classifying patterns.

Based on the mutual information measure returned by MIM for each pattern feature, the *L* pattern features were ranked in descending order of importance. Then the top 1, 2, 4, 6,…,98 and 100% of the ranked *L* pattern features—denoted in the following as *L*$^{s}$—were utilised to construct and test classifiers.

A stratified tenfold cross-validation method was used to validate classifiers. In each fold, nine portions of the data set were utilised as a training data set to construct the classifier employing Eqs. 7, 9, 11, 12 and 14. The remaining 10th portion was used as a test set.

The performances of SNB, LCMNB and TNB classifiers on a given dataset were compared by using McNemar’s test statistics and a multi-class Matthews correlation coefficient (MCC) measure [33].

To generate a TNB classifier, it was necessary to optimize the $\lambda$ parameter (in Eq. 11). In this work, the MCC measure metric and the tenfold cross-validation scheme were employed to optimise the value of $\lambda$.

## Results and discussion

We should state from the outset that the classification results presented in this study were retrospective in the sense that the classes predicted for the test ligands were known beforehand.

Note that, although the *x*-axes in the figures below show the total number of features employed, in the LCMNB case only the presence of features was considered.

### Testing: conditions 1 and 2

#### The enzymes dataset: 4,658 ligands distributed over 31 enzymes

**Figure 1:**
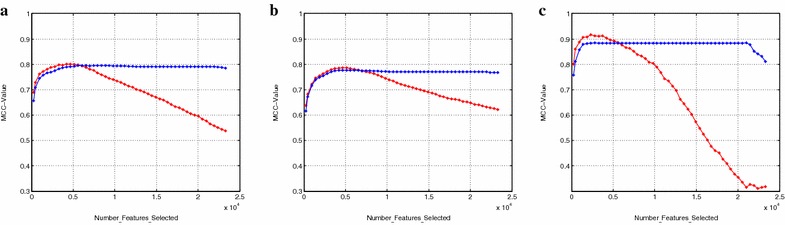
Plots showing the MCC values of the classification performances of the SNB (*red line*) and LCMNB (*blue line*) classifiers versus the number of features employed for the three datasets: **a** enzyme data set; ** b** membrane receptor data set; and** c** mixed class data set.

Figure 1a shows a plot of MCC values returned by the SNB (red line) and LCMNB (blue line) classifiers vs the number of features selected to construct and test the classifiers.

The SNB model performed best when the value of *L*$^{s}$ was 4,665 (i.e., the top 20% of the *L* ranked features were utilised); the MCC value obtained was 0.801. The corresponding MCC value returned by the LCMNB model using this set of features was 0.791. The pair of MCC values were similar, within 1.2% of each other. However, looking into the statistics of the two classification results obtained by the two approaches revealed that at the significance level of 0.05 and one degree of freedom a McNemar’s test yielded a $\chi ^{2}$ value of 4.290. This means the two algorithms performed differently, whereby the SNB classifier outperformed the LCMNB classifier for the data set employed when $L_{s}=4,665$ (see Figure 1a). Furthermore, the SNB classifiers systematically outperformed their LCMNB counterparts whenever *L*_*s*_ < 5,131. However, the two methods performed similarly when the value of $L_{s}$ was between 5,131 and 6,531. When the value of *L*_*s*_ > 6,531, Figure 1a clearly illustrates that the LCMNB approach systematically outperformed the SNB algorithm. In fact, as the value of $L_{s}$ was increased, the classification performance of the SNB model plunged, whereas the classification performance of the LCMNB remained less “sensitive” (in comparison to SNB) to the notably high values of $L^{s}$.

This observation is not surprising and dovetails well with the explanations given in "Methods"—in that section, it was described the reason why the SNB model becomes more biased as the value of *L*$^{s}$ considerably increases beyond the value of *L*$^{s}$ with which the SNB model performs best, based on the the dataset utilised.

#### The receptors dataset: 5,031 ligands annotated against 23 membrane-receptors

Figure 1b depicts the MCC values returned by the SNB (red line) and LCMNB (blue line) classifiers plotted against the number of features utilised, *L*$^{s}$, to define the binary feature space, on which the classifiers were constructed. The SNB classifiers systematically outperformed their LCMNB counterparts whenever *L*_*s*_ ≤ 6,531. The SNB model performed best when $L_{s}$ was equal 5,131 (that is, when the top 22% of the ranked *L* features were employed); the MCC value obtained was 0.786. The corresponding MMC value returned by the LCMNB model based on this set of features was 0.777. The two MCC values are within 1% of each other. However, looking into the statistics of the two classification results obtained by the two models, once again, revealed otherwise: at the significance level of 0.05 and one degree of freedom, a McNemar’s test yielded a $\chi ^{2}$ value of 5.693 in favour of the SNB algorithm. (Here, “in favour” means the number of test ligands misclassified by the SNB model but not the LCMNB model is smaller than the number of test compounds misclassified by the LCMNB model but not the SNB model for the test dataset.) When $L_{s}$ was between 6,064 and 8,397, the two models performed similarly according to McNemar’s test. Figure 1b demonstrates that the LCMNB classifier systematically outperforms its corresponding SNB classifier when the value of *L*_*s*_ > 8,397. This discrepancy became prominent when $L_{s}$ is notably larger than 8,397: the performance of the SNB model markedly deteriorated, while performance of the LCMNB approach barely changed. The reason behind this observation is as explained before.

#### The mixed dataset: 1,149 ligands (four transporters, one transcription factor and one ion-channel)

Figure 1c illustrates a plot of MCC values returned by SNB (red line) and LCMNB (blue line) classifiers against the number of features employed to construct and test these classifiers.

For this dataset, the SNB classifier performed best when the top 2,332—i.e., 10% of the *L*—features were utilised. The MCC value returned was 0.917. The corresponding MCC value yielded by the LCMNB classifier returned was 0.884. Here, at a significance level of 0.05 and one degree of freedom, a McNemar’s test performed on the two sets of classifications results returned by the two models gave a $\chi ^{2}$ value of 17.647 in favour of the SNB approach—in favour in the sense described in the previous section. The two algorithms performed similarly (albeit statistically) when the top number of features employed was not markedly different from 2,332. However, as the top number of features selected drifted away upwards from 2,332, the classification performance of the SNB approach deteriorated, while the LCMNB scheme performance showed lesser “sensitivity” (in comparison to SNB) to significantly increasing the value of *L*$^{s}$, see Figure 1c. The explanation for this phenomenon is as given above.

One immediately observes that the best classification performances for all three data sets were achieved by the SNB approach. Furthermore, these best performances, which were supported by statistical tests, were notably obtained only when particular subsets of the *L* ranked features were employed.

From these analyses, based on our three datasets, we can surmise two main points. First, Conditions 1 and 2 do not always hold. Had they held, the two sets of results would have been (statistically or otherwise) similar. Hence, one should pay careful attention to these facts when it comes to applying LCMNB as a substitute for SNB. Secondly, the best classification performances were returned by the SNB approach for all three data sets. This suggests that taking into account absence of features—provided one does not utilise them in a slipshod manner—can have discriminatory powers, capable of enhancing the classification ability of the SNB algorithm.

In summary, our analyses indicate that feature selection is a better option than severely penalising out absence of features.

### Comparing the LCMNB, SNB and TNB approaches

Here we present what happens when one judiciously combines both feature selection and penalising out absence of features.

For all three data sets, $\lambda$ was varied from 0 to 1. For a dataset, the $\lambda$ value that results in the best MCC value was considered “optimal” for that dataset.

#### The enzymes dataset: 4,658 ligands distributed over 31 enzymes

**Figure 2:**
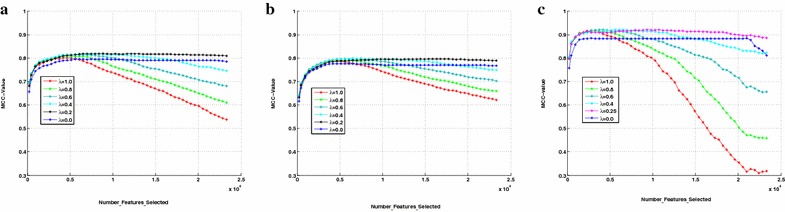
Plots showing the MCC values of the classification performances of TNB classifiers (per $\lambda$ value) versus the number of features employed for the three datasets:** a** enzyme data set;** b** membrane-receptor data set; and** c** mixed class data set.

Figure 2a depicts plots of MCC values returned by TNB vs $L_{s}$ for different $\lambda$ values varied from 0 to 1.0 in steps of 0.2. Neither LCMNB ($\lambda$ = 0.0; blue line) classifiers nor SNB (red line; $\lambda$ = 1.0) classifiers performed best. The best classification performance, MCC value of 0.820, was yielded by a TNB classifier whereby the value of $\lambda$ was 0.2; with $L_{s}$ being equal to 8,863—that is, the top 38% of the total number of features were utilised. Then the value of $\lambda$ was varied in the range [0.05,0.35] in steps of 0.05, but this did not improve on the MCC value yielded by the TNB classifier where the value of $\lambda$ was 0.2.

This TNB classifier systematically outperformed all the other classifiers including those based on SNB and LCMNB. Thus, it was not essential to statistically validate our findings. However, suffice it to say at a significance level of 0.05 and one degree of freedom, a McNemar’s test performed on any two corresponding sets of classifications results returned by TNB and SNB (or LCMNB) gave no $\chi ^{2}$ value smaller than 3.84. This meant that the TNB and SNB (or LCMNB) classifiers performed differently, with the TNB classifier systematically outperforming both SNB and LCMNB classifiers.

For completeness we also looked into the classification performances of the TNB and LCMNB classifiers on classifying test ligands for each of the 31 target proteins in this dataset. The results are shown in Columns 2–3 of Table 4, which indicate that the LCMNB classifier performed similarly or better than the TNB classifier for only five (out of the 31) target proteins, vide the MCC values in italics print in the table.

**Table 4:** Enzyme data set: columns 1 denotes the target identifier

Target ID	$L_{s}$ = 8,863	
	MCC$_{TNB}$	MCC$_{LCMNB}$
10980	0.903	0.869
15	0.424	0.375
11489	0.970	0.963
10193	0.345	0.286
12252	0.980	0.980
11140	0.980	*0.990*
9	0.831	0.806
10188	0.960	0.949
12952	0.539	0.429
194	0.986	0.980
93	0.749	0.746
126	0.973	0.960
12697	0.987	*0.993*
104	0.939	*0.939*
11	0.973	0.963
11225	0.985	0.968
11727	0.928	*0.932*
11024	0.711	0.682
10495	0.871	0.870
11534	0.833	0.819
13001	0.499	0.473
13061	0.981	0.964
12666	0.981	0.967
10532	0.724	0.714
65	0.922	0.912
188	0.701	0.693
10434	0.927	0.813
11451	0.936	0.867
13000	0.784	0.751
10197	0.919	0.887
12209	0.0267	*0.271*

Columns 2 and 3 represent the MCC values obtained by TNB ($\lambda =0.2$) and LCMNB for each of the 31 targets. L$^{s}$ is the number of features employed.

#### The receptors dataset: 5,031 ligands annotated against 23 membrane-receptors

Like for the enzymes data set, Figure 2b shows plots of MCC values returned by TNB vs *L*$^{s}$ for different values of $\lambda$ varied from 0 to 1.0 in steps of 0.2. Here, again, neither LCMNB ($\lambda =0.0$; blue line) classifiers nor SNB ($\lambda =1.0$; red line) classifiers performed best. The best MCC value of 0.797 was achieved by a TNB classifier with the value of $\lambda$ being 0.2, and $L_{s} = 9796$—that is, the top 42% of the total number of features were utilised. The value of $\lambda$ was varied in the range [0.05,0.35] in steps of 0.05, but this did not improve on the MCC value obtained by the TNB classifier where the value of $\lambda$ was 0.2.

We also looked into the classification performances of the TNB and LCMNB classifiers on classifying test ligands for each of the 23 target proteins in this dataset. Columns 2–3 of Table 5 indicate that the LCMNB classifier performed better than the TNB classifier for only two (out of the 23) target proteins, see the MCC values in italics print in the table.

**Table 5:** Membrane-receptor data set: columns 1 denotes the target identifier

Target ID	$L_{s}$ = 9,796	
	MCC$_{TNB}$	MCC$_{LCMNB}$
252	0.857	0.853
280	0.862	0.852
114	0.633	0.579
259	0.823	0.781
10280	0.962	0.959
87	0.803	0.781
72	0.639	0.582
129	0.455	0.430
137	0.525	0.523
136	0.644	*0.645*
10142	0.986	0.981
51	0.794	0.778
130	0.785	0.777
19905	0.971	0.970
10627	0.958	*0.967*
107	0.767	0.759
11575	0.947	0.929
278	0.749	0.717
20174	0.953	0.844
108	0.790	0.780
11290	0.919	0.904
10580	0.941	0.759
138	0.950	0.840

Columns 2 and 3 represent the MCC values obtained by TNB ($\lambda =0.2$) and LCMNB for each of the 23 targets. $L_{s}$ is the number of features utilised.

#### The mixed dataset: 1,149 ligands (four transporters, one transcription factor and one ion-channel)

Figure 2c shows plots of MCC values returned by TNB vs $L_{s}$ for different values of $\lambda$ varied from 0 to 1.0. Once again it was not SNB ($\lambda =1.0$; red line) nor LCMNB ($\lambda =0.0$; blue line) that obtained the best classification results. The best performance was returned by a TNB classifier where the value of $\lambda$ was 0.25, and $L_{s}$ was equal to 9,330—that is, the top 40% of the total number of features were utilised. The MCC value obtained by this TNB classifier was 0.921.

Columns 2–3 of Table 6 show the classification performances of the TNB and LCMNB classifiers on classifying test ligands for each of the six target proteins in the dataset. The two sets of MCC values indicate that the LCMNB classifier performed similarly or better than the TNB classifier for one (out of the six) target proteins as the the MCC values in italics print in the table depict.

**Table 6:** Mixed class data set: columns 1 denotes the target identifier

Target ID	$L_{s}$ = 9,330	
	MCC$_{TNB}$	MCC$_{LCMNB}$
121	0.863	0.838
100	0.827	0.817
155	0.866	*0.886*
20092	1.000	0.995
165	0.975	0.926
133	0.994	0.830

Columns 2 and 3 represent the MCC values obtained by TNB ($\lambda =0.25$) and LCMNB for each of the 6 targets. $L_{s}$ is the number of features utilised.

Now, based on the data sets utilised, we may conclude: combining feature selection with apt penalization of absence of features can improve the classification performance of the Bernoulli Naive Bayes algorithm, in particular when the value of $L_{s}$ is large and the training pattern vectors are highly sparse (in the sense described in “Background”).

## Conclusion

In this work, we set out to examine the validity of a claim made in a paper by Mussa, Mitchell and Glen (MMG) concerning the application of the conceptually simple and computationally efficient classification algorithm, the LCMNB approach of Xia et al. MMG pointed out that the central tenet of the LCMNB approach—ignoring the role of feature absence when utilising Bernoulli Naïve Bayes algorithms for classification purposes—might only be justifiable under certain conditions.

If these conditions hold, LCMNB classifiers were expected to perform similarly to the SNB classifiers on classifying the test data sets employed in this work. However, SNB and LCMNB classifiers performed differently, whereby SNB classifiers returned the best classification results for all the three bioactivity data sets utilised in this study. These results suggest that taking into account—albeit prudently—absence of a feature can enhance (not impair) the classification ability of the SNB approach.

In this work, we also introduced a new variant of the Naïve Bayes algorithm termed “Tapered Naïve Bayes”, which encapsulates both LCMNB and SNB. Constructed and then tested on our three biactivity data sets, TNB systematically outperformed both SNB and LCMNB. These classification results lend support to the simple idea on which TNB was anchored—i.e., in order to avoid ending up with a highly biased Naïve Bayes classifier, when the value of *L* is large and the training pattern vectors are highly sparse (in the sense described before), penalise appropriately the contributions from absence of features to the classifier.
